# Light and dark biofilm adaptation impacts larval settlement in diverse coral species

**DOI:** 10.1186/s40793-025-00670-0

**Published:** 2025-01-25

**Authors:** Paul A. O’Brien, Sara C. Bell, Laura Rix, Abigail C. Turnlund, Shannon R. Kjeldsen, Nicole S. Webster, Andrew P. Negri, Muhammad A. Abdul Wahab, Inka Vanwonterghem

**Affiliations:** 1 Australian Centre for Ecogenomics, School of Chemistry and Molecular Biosciences, The University of Queensland, St Lucia, QLD Australia; 2 Australian Institute of Marine Science, Townsville, QLD Australia; 3 AIMS@JCU, James Cook University, Townsville, QLD Australia; 4 Centre for Sustainable Tropical Fisheries and Aquaculture, James Cook University, Townsville, QLD 4811 Australia; 5 Institute for Marine and Antarctic Studies, University of Tasmania, Hobart, TAS 7001 Australia; 6 Commonwealth Scientific and Industrial Research Organisation, Dutton Park, QLD Australia

**Keywords:** Coral recruitment, Reef restoration, Settlement cues, Biofilms, 16S rRNA gene

## Abstract

**Background:**

Recovery of degraded coral reefs is reliant upon the recruitment of coral larvae, yet the mechanisms behind coral larval settlement are not well understood, especially for non-acroporid species. Biofilms associated with reef substrates, such as coral rubble or crustose coralline algae, can induce coral larval settlement; however, the specific biochemical cues and the microorganisms that produce them remain largely unknown. Here, we assessed larval settlement responses in five non-acroporid broadcast-spawning coral species in the families Merulinidae, Lobophyllidae and Poritidae to biofilms developed in aquaria for either one or two months under light and dark treatments. Biofilms were characterised using 16S rRNA gene sequencing to identify the taxa associated with settlement induction and/or inhibition.

**Results:**

We show that light and biofilm age are critical factors in the development of settlement inducing biofilms, where different biofilm compositions impacted larval settlement behaviour. Further, we show that specific biofilm taxa were either positively or negatively correlated with coral settlement, indicating potential inducers or inhibitors. Although these taxa were generally specific to each coral species, we observed bacteria classified as *Flavobacteriaceae*, *Rhodobacteraceae*, *Rhizobiaceae* and *Pirellulaceae* to be consistently correlated with larval settlement across multiple coral species.

**Conclusions:**

Our work identifies novel microbial groups that significantly influence coral larval settlement, which can be targeted for the discovery of settlement-inducing metabolites for implementation in reef restoration programs. Furthermore, our results reinforce that the biofilm community on coral reef substrates plays a crucial role in influencing coral larval recruitment, thereby impacting the recovery of coral reefs.

**Supplementary Information:**

The online version contains supplementary material available at 10.1186/s40793-025-00670-0.

## Background

Coral recruitment is a vital process in maintaining a healthy and resilient reef ecosystem [1], and facilitating larval recruitment is one of several strategies employed to actively restore degraded reefs [2, 3]. During the recruitment process, competent larvae migrate to the benthos in search of a suitable location to attach and undergo metamorphosis, a process known as larval settlement [4]. A range of (a)biotic cues signal larvae to begin the settlement process by selecting a suitable site for attachment [5]. For example, coral larvae are reported to preferentially settle in response to light intensity and surface topography [6, 7]. However, biochemical cues from benthic sources are the most potent and specific inducers of settlement, with potential applications in coral restoration and conservation aquaculture [2, 5]. Despite this promise, our understanding of the origins of biochemical cues and mechanisms of induction remain poorly understood.

Sources of biochemical settlement cues have been primarily attributed to crustose coralline algae (CCA) and marine biofilms. Corals from both the Acroporidae and non-acroporid families have demonstrated high settlement responses to various species of CCA, although the effectiveness of CCA as an inducer varies substantially among coral-CCA species combinations [8–11]. Similarly, corals settle in response to marine biofilms, including biofilm communities on substrates such as coral rubble or artificial surfaces [12, 13], or single-species biofilms isolated from various reef substrates [14–17]. Chemical compounds extracted from both CCA and biofilms can also induce settlement [18–22]. However, since CCA harbour their own unique surface biofilms [23], it can be difficult to disentangle the source of the settlement cue and differentiate between host and microbe effects. Nonetheless, some settlement cues are thought to be associated with the algal cell wall [24, 25], while others have been extracted from prokaryotes isolated from the CCA surface [19, 20, 26]. Given that most marine invertebrate phyla have representative species that undergo metamorphosis in response to a bacterial cue [27], it is likely that bacteria-induced larval settlement is common amongst coral species.

Settlement cues for marine larvae that originate from bacteria are diverse and range from chemical (either surface-bound or soluble) to mechanical (e.g. injection systems) [22, 27–29]. Perhaps the most comprehensively characterised system is the bacteria-induced settlement of the tubeworm, *Hydroides elegans*, by *Pseudoalteromonas luteoviolaceae.* Here, phage tail-like structures inject an effector protein (*Mif1*) into the larvae which stimulates metamorphosis [30, 31]. In a very different process, multiple strains of *Pseudoalteromonas* sp*.* can stimulate the metamorphosis of a variety of scleractinian corals through biosynthesis of the brominated aromatic hydrocarbon, tetrabromopyrrole (TBP), [20, 32–34]. However, in some coral species TBP-induced metamorphosis can occur without larval attachment and, therefore, TBP is not an ideal candidate as a biochemical inducer for coral aquaculture or restoration applications [18, 20]. Furthermore, *Pseudoalteromonas* spp. are not commonly found in ecologically relevant abundances in natural reef biofilms, so *Pseudoalteromonas-*derived TBP is unlikely to be solely responsible for larval settlement on the reef [18].

An alternative mechanism of bacteria-induced settlement was recently reported, where the pigment cycloprodigiosin, isolated from *Pseudoalteromonas rubra*, reliably induced settlement of *Leptastrea purpurea* larvae [22, 26]. In this light-dependent reaction, cycloprodigiosin molecules, which are harvested by coral larvae, undergo photodegradation to continuously produce hydrogen peroxide (H_2_O_2_), a compound believed to stimulate metamorphosis [26]. However, because cycloprodigiosin is unstable in the presence of oxygen and light, and because H_2_O_2_ alone can induce metamorphosis without promoting attachment [26], using cycloprodigiosin on a larger scale for coral restoration would be challenging. Nonetheless, this research highlights that novel mechanisms for settlement induction could lead to the development of reliable treatments to control larval settlement in aquaculture. Investigating uncultured biofilm communities associated with larval settlement has high potential for identifying new microbial candidates to achieve this goal.

While evidence points towards CCA as a primary source of biochemical inducers for settlement of acroporid larvae [9, 18], microbial sources of biochemical inducers are likely to play a role in settlement of some non-acroporid coral larvae [26, 34]. Marine biofilms offer a diversity of potential prokaryotic and eukaryotic sources of inductive biochemistry, although teasing apart specific cues within these highly diverse and complex biofilm communities can be challenging. The establishment of biofilms in darkness is likely to result in dominance of non-phototrophic taxa and may help identify which prokaryotes are potential sources. Therefore, we assessed the influence of light and dark conditions on the development of prokaryote biofilm communities and linked these communities to settlement behaviour in larvae from five species of non-acroporid broadcast-spawning coral. We focus on prokaryotes as our main goal is to identify new target groups that can be cultured and investigated for biochemical applications in coral larval settlement. Moreover, prokaryotes have an enormous metabolic capability with wide-ranging possibilities for mechanisms of settlement induction [29]. Firstly, we taxonomically characterised biofilms that induce larval settlement across the range of coral species. Secondly, we tested whether extractable compounds associated with these biofilms also induce larval settlement. Finally, we identified prokaryotes that are associated with high or low larval settlement across multiple coral species, highlighting their potential to induce or inhibit settlement more broadly.

## Methods

### Substrate conditioning for biofilm development and chemical extraction of biofilms

Biofilm development was undertaken at the National Sea Simulator (SeaSim) at the Australian Institute of Marine Science (AIMS) in Townsville, QLD, Australia. Biofilms were formed on concrete substrate sheets (Figure S1), that were designed within the Reef Restoration and Adaptation Program (RRAP) as an experimental substrate for coral larval settlement in aquaculture [3, 35]. Each sheet was constructed to enable the generation of smaller tabs (herein referred to as settlement tabs) measuring 14 × 14 mm (n = 100), by breaking them off along grooves that allow for clean fractures (Figure S1). Conditioning occurred separately under both light and dark treatments to understand if light is necessary for the formation of settlement-inducing biofilms. Additionally, biofilms were developed for two time periods, 1 month (1 M) and 2 months (2 M), under both treatment conditions, to observe the succession in the biofilm community and understand if this corresponds to larval settlement success.

Conditioning occurred in 48 L acrylic experimental tanks placed inside a 275 L fiberglass tank that contained mixed reef substrate (including coral rubble, benthic algae, and live coral) as a source of natural microbial diversity to seed the experimental biofilms (Figure S1). Seawater temperature was profiled to daily averages recorded at Davies Reef, central Great Barrier Reef (GBR) from 1998 to 2015 (range 23.7‒27.3 °C). The fiberglass tanks form part of a semi-recirculating system that received new input of 1 µm filtered seawater (FSW) into a sump at a rate of ~ 3 turnovers per day. Seawater was circulated from the fiberglass tank to the sump and back through the smaller experimental tanks, with flow rates into the experimental tanks set to 1.2–1.3 L min^−1^, equivalent to ~ 36 turnovers day^−1^ to allow for sufficient seeding of biofilms. Dark treatment tanks were covered in black plastic Corflute sheeting to ensure no light reached the settlement sheets (Figure S1). Light treatment sheets were exposed to custom 300 W panel LED lights with a photoperiod set to follow the local (Townsville, QLD, Australia) sunrise and sunset times and a peak intensity of approximately 400 µmol photons m^−2^ s^−1^ photosynthetically active radiation (PAR). Each light and dark treatment was replicated in three independent tank systems, yielding a total of twelve treatment x tank combinations of biofilm development.

Negative controls consisted of unconditioned concrete settlement tabs that had been soaked in 0.1 µm FSW for approximately 1 week, refreshed 2–3 times and sterilised by autoclave. At the end of the conditioning period, a subset of 2 M light settlement tabs were removed for chemical extraction using two solvents: ethanol (EtOH) for polar compounds and dichloromethane (DCM) for hydrophobic compounds. The extraction aimed to test the efficacy of the different compound solubility classes in inducing settlement and to determine whether biofilm chemistry alone could induce settlement without surface topography. The remainder were held in conditioning tanks until set-up of settlement assays to test the direct settlement of larvae on biofilms. Full details of chemical extraction methods can be found in Supplemental File 1.

### Coral collection, spawning and larval rearing

Larval cultures used for this experiment are described in Abdul Wahab et al., (2023), detailing methods for coral collection, spawning and larval rearing. Briefly, spawning experiments were conducted during the 2021 Great Barrier Reef (GBR) mass spawning events following the October and November full moons (Table 1). Coral colonies from the families Merulinidae (*Platygyra sinensis* and *Dipsastrea favus*), Lobophyllidae (*Echinophyllia aspera* and *Lobophyllia corymbosa*)*,* and Poritidae (*Porites lobata*) were collected approximately 1 week before the predicted spawning event and brought to SeaSim AIMS where they were held in outdoor semi-recirculating aquaria. Colonies were isolated into 60 L tanks for gamete collection and gametes from all parent colonies of the same species were pooled for cross-fertilisation. Embryos were rinsed and transferred to 70 L flow-through tanks where larval cultures were maintained until used in settlement assays.

**Table 1:** Details of corals spawned over the October–November 2021 mass spawning on the central Great Barrier Reef

Coral species	# Parent colonies	Spawning event	Collection location	Spawning date	Assay prep date	Larval age
*Dipsastrea favus*	4	October	Magnetic Island	23-10-2021	30-10-2021	7 days
*Platygyra sinensis*	2	October	Magnetic Island	24-10-2021	29-10-2021	5 days
*Echinophyllia aspera*	4	October	Magnetic Island	28-10-2021	04-11-2021	7 days
*Porites lobata*	5	November	Palm Island Group	25-11-2021	30-11-2021	5 days
*Lobophyllia corymbosa*	6	November	Palm Island Group	26-11-2021	04-12-2021	8 days

### Coral larval settlement assays

Larval competency tests were performed 5–7 days after fertilisation using a range of settlement substrates including the CCA *Porolithon* sp., 2 M light conditioned tabs and a combination of both. Larvae were considered competent for use in assays after they had demonstrated > 80% settlement in response to at least one substrate type. Settlement assays were prepared using sterile 6-well culture plates (Corning Costar TC-Treated, Merck) in a temperature-controlled room (27–28 °C) with a light photoperiod set to follow the local (Townsville, QLD, Australia) sunrise and sunset times. Each well was first filled with 10 mL of 0.1 µm FSW, larvae added (n = 6), followed by the settlement tab. Each plate contained five replicate wells of a conditioning treatment and one negative control. Six replicate plates of each tank (n = 3) and treatment (n = 4) combination were included for a total of 72 plates tested per coral species. Assays were assessed after ~ 48 h and larvae were scored as settled if they were firmly attached to either the substrate or well and showing signs of metamorphosis (Figure S2) [36]. Fluorescence was used to assist in the detection of larvae, using a stereo microscope fluorescence adaptor (https:// night sea. com/; SFA RB—excitation 440–460 nm, emission filter 500 nm longpass) that excites the larval green fluorescent proteins.

For chemical extract trials (November spawning corals only; Table 1), each extract was screened across five volumes in duplicate wells; 5, 10, 25, 50 and 100 µL along with a negative control (0 µL) for a total of 2 × plates per extract. Assays were conducted by first adding the extract directly to the well plate and allowing the solvent to evaporate before the addition of 10 mL FSW and finally larvae (n = 6). Settlement was assessed after ~ 48 h using the criteria above (Figure S2).

### Statistical analysis of settlement assays

To determine the best model to fit the settlement data, we tested a generalised linear model (GLM) and a generalised linear mixed effects model (GLMM), with binomial and quasibinomial distributions, with and without random effects. The best model was chosen by assessing the fit to the residuals and Akaike Information Criterion (AIC) comparison. A GLMM with a binomial distribution was subsequently used to test for differences in the frequency of larval settlement among treatments, including both tank and observation-level random effects. This was used as our data had two outcomes, i.e., settled and not settled, and each observation recorded the proportion of each outcome. Post hoc analyses were conducted using a Tukey’s test to identify differences in larval settlement between each treatment combination, with a Bonferroni correction for multiple comparisons. Similarly, a GLM with a binomial distribution was used to examine differences in larval settlement in response to EtOH and DCM chemical extracts, with responses across all dilutions grouped for comparison. All statistical analyses and figures were completed in RStudio [37] using the packages ‘tidyverse’ [38], ‘lme4’ [39] and ‘multcomp’ [40].

### Sampling of biofilms, coral larvae, and seawater

Following settlement assays each settlement tab was wrapped in sterile aluminium foil, placed into a Whirl–Pak® bag (grouped by treatment) and snap frozen in liquid nitrogen. Additionally, three replicate samples of coral larvae per species were concentrated in a cryovial, seawater removed by pipette and snap frozen in liquid nitrogen to ensure any larvae-associated microbes could be identified. Finally, seawater from both the experimental tanks and FSW used for settlement assays was sampled for comparison to the biofilm community. For the experimental tanks, 5 L of seawater was collected from each tank during biofilm development before each spawning event (on the 22nd September and 29th October). For FSW used in settlement assays, 5 L of FSW was collected during the experiment from the 29th of October until the 1st of November. Seawater samples were processed by filtering through a 0.22 µm Sterivex filter (SVGP01050) using a peristaltic pump and snap frozen in liquid nitrogen. All samples were stored at − 75 °C until further analysis.

A subset of biofilm samples was selected for further microbial community analysis based on the results of larval settlement assays. These included 2 M light treatment biofilms used for all coral species except *L. corymbosa* (n = 53–58 per species), based on the distribution of high to low settlement (see results). Additionally, 1 M light (n = 52) and 2 M dark (n = 42) biofilms from *E. aspera* settlement assays were included to investigate differences in biofilm community between light and dark treatments and biofilm age. 1 M dark biofilms were not included since no difference was observed in settlement results between the 1 M and 2 M time periods for the dark treatment, and *E. aspera* was selected over other corals as it was the only species that showed a significant settlement response to the dark treatment biofilms (see results). Finally, *L. corymbosa* was omitted from further analysis given the lack of response to biofilm settlement cues in this study.

### DNA extraction and sequencing

To prepare biofilms for extraction, settlement tabs were thawed on ice and individual tabs placed in a 50 mL Falcon tube containing 5 mL of cell separation buffer (Supplementary File 1). Settlement tabs were then incubated at room temperature for 30 min with gentle rotation followed by sonication for 10 min at 40 kHz in a sonication bath to dissociate the biofilm from the substrate. The resulting supernatant was mixed by inversion and a 1 mL aliquot was preserved for 16S rRNA gene sequencing by pelleting cells at 16,000 × g for 10 min, removing the supernatant, snap freezing in liquid nitrogen and storing at − 75 °C. DNA from processed biofilms and larval samples was extracted using the DNeasy UltraClean Microbial Kit (Qiagen) following the manufacturer’s protocol, while seawater samples were extracted using a Phenol:Chlorofom:Isoamyl Alcohol protocol. 16S rRNA amplicon sequencing (2 × 300 bp) was conducted at the Australian Centre for Ecogenomics (ACE) on the Illumina MiSeq (v3) platform. Further details on DNA extraction and sequencing can be found in Supplemental file 1.

### Bioinformatic analysis

Raw sequences were imported into QIIME2 v2022.8 [41] for pre-processing and clustering into amplicon sequence variants (ASVs). Briefly, the cut-adapt [42] plug-in was used to remove primer sequences followed by denoising using the DADA2 [43] plug-in to remove low quality reads, merge paired ends and pick representative ASVs. Taxonomic assignment of ASVs was conducted using the Naïve Bayes classifier pre-trained on the Silva 138 99% OTU database customised to the V4 region using the primers above (see DNA extraction and sequencing). The resulting ASV count table and taxonomic classifications were imported into RStudio [37] for analysis with extensive use of the packages ‘tidyverse’ [38], ‘vegan’ [44], ‘car’ [45], ‘multcomp’ [40], ‘indicspecies’ [46], ‘Maaslin2’ [47] and ‘randomForest’ [48].

### Statistical analysis of ASVs

ASVs were first filtered to remove any sequences classified as Chloroplast, Mitochondria or Eukaryotes. Raw counts were transformed to relative abundances for taxonomic profiling, where phylum and family level classifications were grouped to the top 20 most abundant taxa across all samples and visualised using stacked bar plots. To understand how conditioning treatment impacted biofilm alpha diversity, ASV richness and Shannon Diversity Index was calculated on the ASV count table normalised by rarefaction to 4000 reads. Significance testing was performed using analysis of variance (ANOVA) on fourth root transformed data to improve assumptions of normality and heteroscedasticity, followed by a post-hoc Tukey’s test with a Bonferroni correction. Beta diversity was assessed using non-metric multidimensional scaling (NMDS) based on a Bray–Curtis dissimilarity matrix, calculated from the non-rarefied ASV table after applying a square root transformation and Wisconsin double standardisation. Significance testing was performed on the standardised dissimilarity matrix using a permutational multivariate ANOVA (PERMANOVA). We tested multiple factors including treatment, conditioning tank, coral species and settlement, where settlement was grouped into categories of high (> 70%; 5–6 settled larvae), medium (35–70%; 3–4 settled larvae) and low settlement (< 35%; 0–2 settled larvae), to understand how the microbial community differed among biofilm samples throughout our experiment.

To identify ASVs that correlated with either high or low settlement (and therefore indicate potential inducers or inhibitors of coral larval settlement), three statistical methods were used, 1) indicator species analysis (IS; ‘indicspecies’ package), used to identify ASVs associated to particular settlement categories (described above), 2) multivariate linear models (LM; ‘Maaslin2’ package) to identify ASVs with positive and negative correlations with coral settlement, and 3) a random forest analysis (RF; ‘randomForest’ package) to identify ASVs with the highest predictive ability for coral settlement. To remove the effect of treatment, only the 2 M light biofilms were used as these showed the strongest settlement response yet still included samples of little or no settlement. Each coral species was tested individually given the potential for different settlement preferences across species [8]. Count data was normalised using total sum scaling (TSS) for both IS and LM analyses, while data for the LM was additionally log transformed to improve linearity. For IS analysis, settlement categories were used as above and results were filtered at 0.5 *A*/*B* values to retain ASVs of interest (where *A* = probability the indicator ASV is found only in the target settlement category, and *B* = probability of finding the ASV in all replicates of the target settlement category). For the LM and RF analyses, data was filtered to remove ASVs with less than 0.1% relative abundance or less than 10% prevalence across all samples and analyses were run using settlement values as a continuous variable, with the experimental tank included as a random effect for the LM. To identify ASVs of interest, LM results were filtered to retain significant (*p* < 0.05) positive or negative correlations with a false discovery rate of 0.25 following a Benjamini–Hochberg correction for multiple hypothesis testing. For the RF analysis, the top 20 ASVs that contributed to the model’s predictive ability (model importance) to classify larval settlement were retained and combined with the ASVs identified from the LM and IS analyses to give a final set of potential settlement inducing and inhibiting ASVs. Lastly, identified ASVs were checked against control samples to ensure positive correlations with settlement were not a result of increased ASVs from settled larvae or artifacts of contamination.

## Results

### Coral larval settlement on light and dark treatment biofilms

**Fig. 1:**
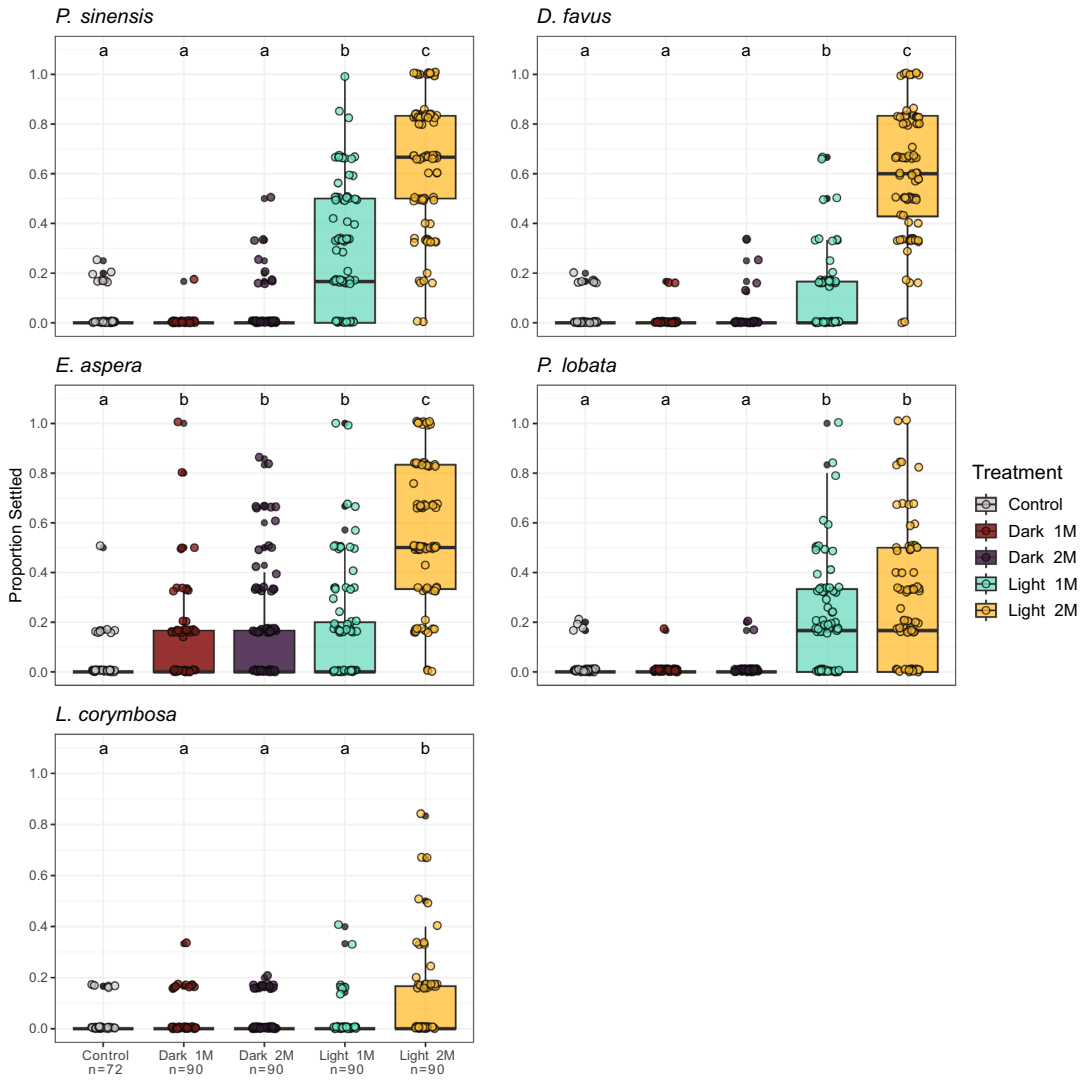
Proportion of coral larvae settled in response to light and dark conditioned biofilms. Control samples represent unconditioned settlement tabs. Letters above boxplots indicate which treatments were significantly different (*p* < 0.05) from each other following a post hoc Tukey’s test. n values indicate the number of replicates in each treatment. 1 M and 2 M indicate one month and two months biofilm conditioning duration respectively

Very low settlement (mean ≤ 2%) was observed for all species on unconditioned control tabs (Fig. 1; Table S1). In contrast, all five coral species settled in response to the biofilms formed on settlement tabs, with settlement consistently highest in the 2-month (2 M) light treatment (Fig. 1; Table S1). Both corals within the family Merulinidae (*P. sinensis* & *D. favus*) had the strongest positive response to settlement cues in this treatment, with a mean settlement of 63.6% (± 27.6 SD) and 59.9% (± 24.7 SD) respectively, while *E. aspera* had a mean settlement of 54.4% (± 29.4 SD) for this treatment. *P. lobata* and *L. corymbosa* showed the weakest response to the 2 M light biofilms, with a mean settlement of 26.1% (± 26.8 SD) and 9.3% (± 16.8 SD) respectively. For the dark treatment biofilms, all coral species except *E. aspera* showed no significant settlement response (GLMM, *p* ≥ 0.05) compared to control settlement tabs. Interestingly, *E. aspera* had a significant settlement response to every treatment compared to control tabs (GLMM, *p* ≤ 0.003; Fig. 1; Table S1). Taken together, both light exposure and conditioning duration had a significant effect on developing biofilms suitable for coral settlement.

During the November settlement assays, chemical extracts of 2 M light conditioned tabs were tested in addition to live biofilms. Here, *P. lobata* demonstrated a stronger response to the dichloromethane (DCM) extract compared to the ethanol (EtOH) extract (GLM; *p* < 0.05; Figure S3), indicating that non-polar compounds within the biofilm may play a role in inducing larval settlement in this species. Furthermore, DCM extract volumes of 10, 25 and 50 µL yielded at least 50% larval settlement, with a maximum of 83% settlement using 25 µL of extract. In contrast, *L. corymbosa* showed no settlement response to either DCM or EtOH extract, consistent with the low settlement induction of the 2 M light biofilms for this species.

### Taxonomic profile of biofilms developed under different conditioning treatments

**Fig. 2:**
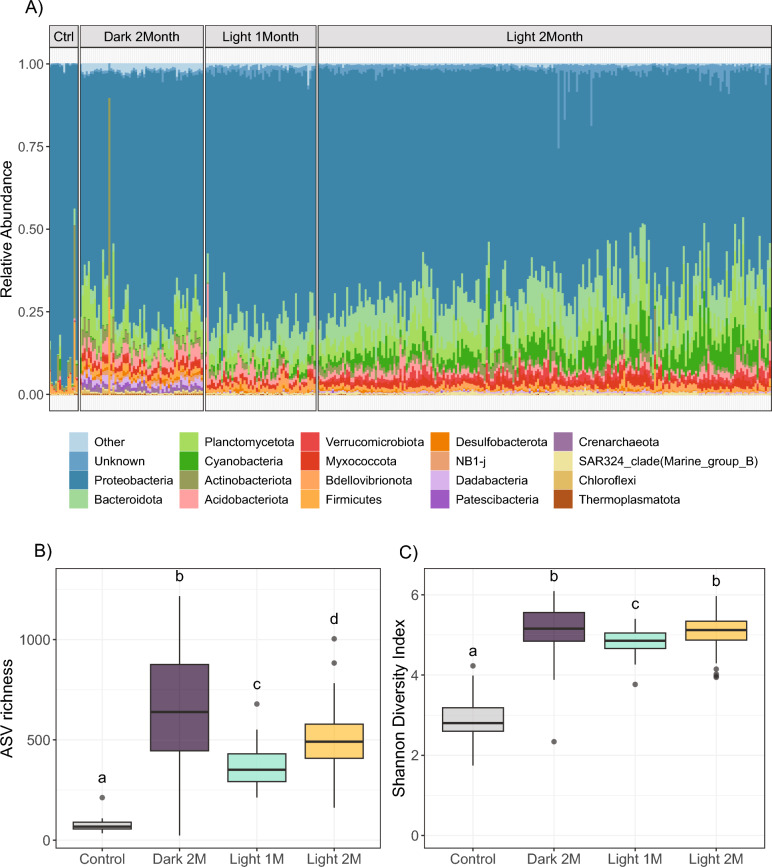
Relative abundance of the top 20 most abundant prokaryote phyla **A**. All taxa not included in the top 20 are grouped as “Other”. Panels **B** and **C** represent ASV richness and Shannon Diversity Index for biofilms in each conditioning treatment respectively. Letters denote which treatments were significantly different from each other (*p* < 0.05). Control samples represent unconditioned settlement tabs while 1 M and 2 M indicate one month and two months biofilm conditioning duration respectively

A total of 13,056,808 reads were recovered across all samples, with a minimum and maximum of 3,205 and 275,207 reads per sample respectively (excluding blanks and controls). Following quality filtering, the total number of reads was reduced to 10,511,754, with a minimum and maximum of 2,497 and 243,645 reads per sample, equating to 36,128 unique ASVs across the dataset. Rarefaction analysis demonstrated that all samples reached asymptote, except for two samples with less than 4000 reads (2 M light treatment) that were subsequently removed from the analysis (Figure S4). Taxonomic profiles based on the 16S rRNA gene showed biofilm communities were extremely diverse with 69 prokaryotic phyla represented across all treatments. While the most abundant phyla were represented across conditioning treatments, differences in their relative abundances were observed (Fig. 2a, S5a). For example, *Cyanobacteria* and *Bacteroidota* had a higher relative abundance in the light treatment biofilms compared to dark biofilms. On the other hand, *Crenarchaeota* and *Dadabacteria* were common in dark treatment biofilms but nearly absent from light treatment biofilms (Supplementary file 2).

At the family level, 525 families were identified with *Rhodobacteraceae* the most abundant across all treatments, followed by *Methyloligellaceae* and *Rhizobiaceae* (Figure S5b). As seen at the phylum level, the most abundant families were found across all treatments with differences observed in their relative abundances. For example, the *Flavobacteraceae* were more abundant in biofilms from the 2 M light treatment compared to both 1 M light and 2 M dark treatment. Further details of taxonomic breakdowns between treatments can be found in Supplementary file 2.

### Alpha and beta diversity of biofilm communities across conditioning treatments

Within sample diversity was influenced primarily by conditioning duration, where more mature biofilms were characterised by a higher diversity (Fig. 2b, c; Figure S6). Although dark treatment biofilms had a higher ASV richness compared to light treatment biofilms (ANOVA; *p* < 0.05), there was no difference in Shannon Diversity Index (*H*) between 2 M dark and 2 M light biofilms (ANOVA; *p* > 0.05; Supplementary file 2). This suggests dark treatment biofilms likely contain a larger number of rare ASVs with low abundances compared to light treatment biofilms.

**Fig. 3:**
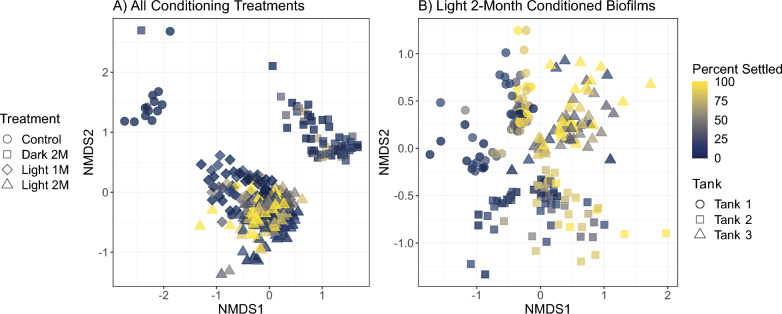
Bray–Curtis dissimilarity among biofilm samples visualised using NMDS. Biofilm samples are coloured by percentage of larvae settled, while shape indicates conditioning treatment **A** or conditioning tank **B**. Light 2-month (2 M) samples clustered together in panel **A** are investigated further in panel **B**, illustrating a gradient of low to high settlement biofilms from left to right

Between sample variability in community composition revealed a strong clustering of biofilm samples by conditioning treatment (PERMANOVA; *F* = 13.4; *p* < 0.001; Fig. 3a, S7; Table S2). Hence, conditioning treatment resulted in biofilms with different community compositions, which correlated to significant changes in larval settlement behaviour. PERMANOVA results also identified a significant relationship between settlement categories (High > 70%, Med 35–70%, low < 35%) and biofilm community composition (*F* = 2.3; *p* < 0.001; Table S2); however, this was not as strong as treatment. Furthermore, we found a significant interaction between settlement category and conditioning treatment (*F* = 1.2; *p* < 0.001; Table S2), suggesting the relationship between settlement category and biofilm composition varied across treatments. Since 2 M light biofilms had the highest success in inducing larval settlement, we additionally investigated variations in community composition within this treatment. Here, patterns of community changes from high-to-low settlement biofilms were observed (Fig. 3b), however the largest effect on biofilm composition was conditioning tank (*F* = 11.3; *p* < 0.001; Table S3), which had a significant interaction with settlement (*F* = 1.5; *p* < 0.001; Table S3). Therefore, although we found a small relationship between coral larval settlement and biofilm community composition, these were dependent on which treatment or conditioning tank biofilms were developed in.

### Microbes associated with high or low coral larval settlement

**Fig. 4:**
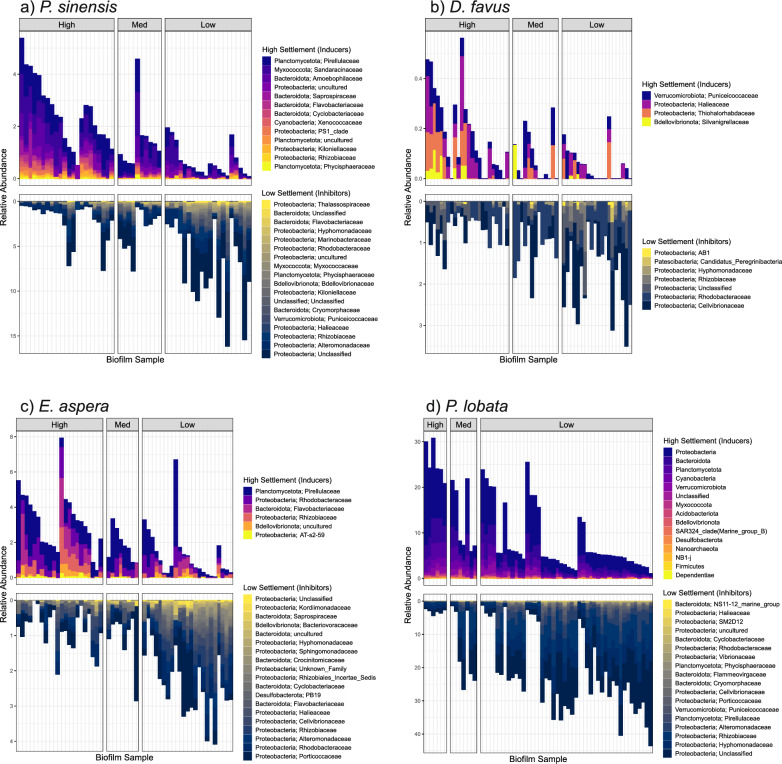
ASVs that correlate with either high or low coral larval settlement within the 2-month light treatment for **a** *Platygyra sinensis*, **b** *Dipsastrea favus,* **c** *Echinophyllia aspera*, and **d** *Porites lobata.* Each bar represents a biofilm sample with the combined relative abundance of ASVs that correlate with high (top panel) or low (bottom panel) settlement. Biofilm samples are ordered by settlement then relative abundance and grouped by High (> 70%), Medium (35–70%) and Low (0–35%) settlement samples. Bars are coloured by ASV family level classification, except for *P. lobata* high settlement ASVs, which are coloured by phylum due to the high number of family classifications

**Fig. 5:**
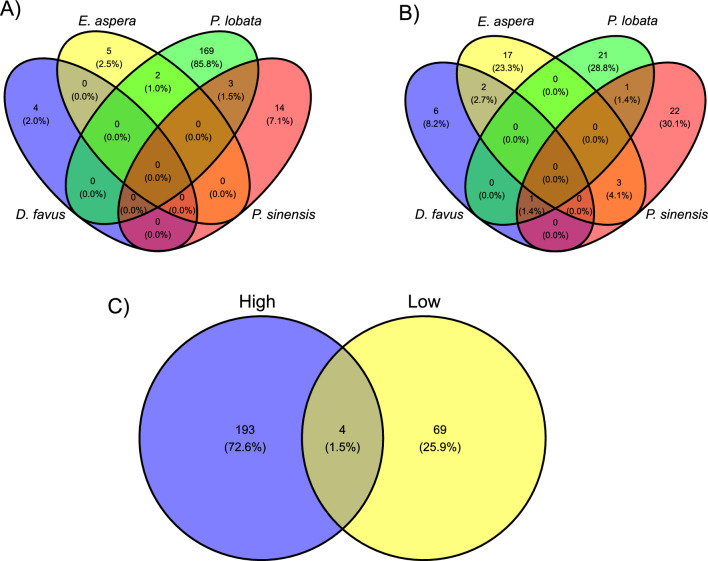
Number of ASVs that are either unique or shared for each coral species tested for **A**) ASVs associated with high settlement, and **B** ASVs associated with low settlement. **C** depicts the total number of ASVs identified across all coral species that were associated with either high or low settlement

To better understand which microbial species within the biofilm community might be driving larval settlement for each coral species, we performed an indicator species (IS) analysis, multivariate linear models (LM) and a random forests (RF) analysis on the 2 M light biofilm samples. Across the three analyses, we found a total of 197 ASVs to be associated with high settlement, where most were associated with *P. lobata* settlement (174 ASVs), while the remaining corals had less than 20 high settlement ASVs each (Figs. 4 and 5; Tables S4-S7). Conversely, we found a total of 73 ASVs associated with low coral settlement, which ranged from 9–27 ASVs for each coral species (Figs. 4 and 5; Tables S4-S7). High settlement ASVs were taxonomically diverse, encompassing a total of 15 phyla and 74 families across all corals. ASVs associated with high *P. lobata* settlement were found across all phyla as well as 70 of 74 families, while ASVs associated with high settlement of the remaining corals were found across 3–5 phyla and 4–12 families. For low settlement, we found ASVs in nine phyla and 34 families in total across all corals, with each species associated with ASVs from 2–7 phyla and 7–19 families.

The relative abundance of ASVs associated with settlement varied across coral species. Combined totals of ASVs associated with high settlement reached greater than 30% of the community in biofilm samples for *P. lobata* assays, but < 1% relative abundance in biofilms for *D. favus* assays (Fig. 4). Similarly, the combined total of ASVs associated with low settlement reached greater than 40% of the biofilm community for *P. lobata* assays, while making up less the 4% of the biofilm community in *D. favus* assays. Of these ASVs, we found very little overlap for those associated with settlement among different coral species, where only 5 ASVs were shared between two corals for high settlement, and no ASVs were shared among three or more corals (Fig. 5; Table 2). Similarly, only 6 ASVs associated with low settlement were shared between two corals, and 1 ASV was shared among three corals (Fig. 5; Table 2). Taken together, these results suggest that the coral settlement response to marine biofilms is species-specific.

**Table 2:** Shared ASVs between corals for high and low settlement. Taxonomy string represents the highest classified level

Settlement	Shared Coral Species	ASV ID	Phylum	Class	Order	Family	Genus
High	*P. lobata* & *E. aspera*	asv_34	*Proteobacteria*	*Alphaproteobacteria*	*Rhodobacterales*	*Rhodobacteraceae*	*Ruegeria*
High	*P. lobata* & *E. aspera*	asv_764	*Proteobacteria*	*Gammaproteobacteria*	*AT-s2-59*	*AT-s2-59*	AT-s2-59
High	*P. lobata* & *P.sinensis*	asv_397	*Planctomycetota*	*Planctomycetes*	*Pirellulales*	*Pirellulaceae*	*Rhodopirellula*
High	*P. lobata* & *P.sinensis*	asv_200	*Proteobacteria*	*Alphaproteobacteria*	*Thalassobaculales*	Unclassified	Unclassified
High	*P. lobata* & *P.sinensis*	asv_84	*Planctomycetota*	*Planctomycetes*	*Pirellulales*	*Pirellulaceae*	*Rubripirellula*
Low	*E. aspera* & *D. favus*	asv_190	*Proteobacteria*	*Alphaproteobacteria*	*Rhizobiales*	*Rhizobiaceae*	Unclassified
Low	*E. aspera* & *D. favus*	asv_110	*Proteobacteria*	*Gammaproteobacteria*	*Cellvibrionales*	*Cellvibrionaceae*	Unclassified
Low	*E. aspera* & *P. sinensis*	asv_452	*Proteobacteria*	*Gammaproteobacteria*	*Alteromonadales*	*Alteromonadaceae*	*Aliiglaciecola*
Low	*E. aspera* & *P. sinensis*	asv_120	*Proteobacteria*	*Gammaproteobacteria*	*Cellvibrionales*	*Halieaceae*	*Pseudohaliea*
Low	*E. aspera* & *P. sinensis*	asv_58	*Proteobacteria*	*Gammaproteobacteria*	*Alteromonadales*	*Alteromonadaceae*	*Aestuariibacter*
Low	*P. lobata* & *P.sinensis*	asv_270	*Bacteroidota*	*Bacteroidia*	*Flavobacteriales*	*Cryomorphaceae*	Unclassified
Low	*P. lobata* & *P.sinensis* & *D. favus*	asv_33	*Proteobacteria*	*Alphaproteobacteria*	Unclassified	Unclassified	Unclassified
High–Low	*P. lobata* (H) & *P.sinensis* (L)	asv_316	*Planctomycetota*	*Phycisphaerae*	*Phycisphaerales*	*Phycisphaeraceae*	*Algisphaera*
High–Low	*P. lobata* (H) & *P.sinensis* (L)	asv_7	*Proteobacteria*	*Gammaproteobacteria*	*Alteromonadales*	*Alteromonadaceae*	*Alteromonas*
High–Low	*P. lobata* (H) & *P.sinensis* (L)	asv_167	*Verrucomicrobiota*	*Verrucomicrobiae*	*Opitutales*	*Puniceicoccaceae*	*Verruc-01*
High–Low	*P. lobata* (H) & *E. aspera* (L)	asv_655	*Bacteroidota*	*Bacteroidia*	*Chitinophagales*	*Saprospiraceae*	*Lewinella*

While no single ASV was significantly correlated with high or low settlement for all coral species tested, when considering higher taxonomic levels, ASVs of the same family were observed to correlate with settlement across the different coral species (Fig. 4). For example, ASVs classified as *Flavobacteriaceae* (*Bacteroidota*), *Pirellulaceae* (*Planctomycetota*) and *Rhizobiaceae* (*Proteobacteria*) were all associated with high settlement for the coral species *P. sinensis*, *E. aspera* and *P. lobata* (Fig. 4a, c, d), while *Rhodobacteraceae* (*Proteobacteria*) ASVs additionally correlated with high settlement of *E. aspera* and *P. lobata* (Fig. 4c, d). Although not all genera within these families were classified (Table S4-S7), those that were showed consistencies and disparities in which taxa correlated with settlement across different coral species. For example, within *Flavobacteriaceae*, the genus *Flagellimonas* was associated with high settlement of *P. sinensis*, while the genera *Aquimarina* and *Muricauda* were associated with high settlement of *P. lobata*. On the other hand, within *Pirellulaceae*, the genera *Blastopirellula* and *Rhodopirellula* were associated with high settlement of all three corals, while *Rubripirellula* was associated with high settlement of *P. sinensis* and *P. lobata*. Similarly, the genus *Ruegeria* within *Rhodobacteraceae* was associated with high settlement of both *E. aspera* and *P. lobata*, while another six identified genera associated with high *P. lobata* settlement (Table S7), including *Roseivivax.*

Interestingly, other ASVs within these families correlated with low coral settlement, highlighting that some closely related bacterial species may elicit different settlement responses. Here, *Rhizobiaceae* and *Rhodobacteraceae* ASVs correlated with low coral settlement in all species tested (Fig. 4), while *Flavobacteriaceae* ASVs were correlated with low coral settlement for *P. sinensis* and *E. aspera* (Fig. 4a, c)*.* Similarly, ASVs classified as *Pirellulaceae* correlated with low coral settlement, however this was only seen in *P. lobata* (Fig. 4d)*.* Within these families, we identified some genera that were associated with high and low settlement. For example, *Winogradskyella* (*Flavobacteriaceae*) was associated high settlement of *P. lobata* and low settlement of *P. sinensis*, while *Pir4* (*Pirellulaceae*) was associated high settlement of *P. sinensis* and both high and low settlement of *P. lobata.* Similarly, *Limibaculum* (*Rhodobacteraceae*) was associated with high and low settlement of *P. lobata*. However, by and large, most genera associated with high settlement were not associated with low settlement.

In addition to those bacterial families that contained both high and low settlement associated ASVs, we also identified families that predominantly contained ASVs associated with low coral settlement. For example, ASVs classified as *Hyphomonadaceae* (*Proteobacteria*) were associated with low coral settlement in all species (Fig. 4), while ASVs classified as *Alteromonadaceae* (*Proteobacteria*) were associated with low settlement in *P. sinensis, E. aspera* and *P. lobata* (Fig. 4a,c,d). Similarly, ASVs classified as *Cellvibrionaceae* (*Proteobacteria*) were associated with low settlement in *E. aspera*, *P. lobata* and *D. favus* (Fig. 4b,c,d). Moreover, these three families were not associated with high settlement for any coral except *P. lobata,* with *Alteromonas* being the only genus observed in high and low settlement biofilms, suggesting these families may have an inhibitory effect on coral larval settlement.

We also investigated whether the ASVs that correlated with high or low settlement for each coral in the 2 M light treatment were also present in the treatments that did not induce a high amount of larval settlement, including the 1 M light, 2 M dark and control treatments (Table S8). The presence of potential settlement inducing and inhibitory ASVs in all treatments suggests it is the relative abundance of microbes within the community, along with the complexity of their interactions, that govern the settlement inducing ability of biofilms, rather than presence/absence of specific taxa alone. Further, we note that none of the potential settlement inducing ASVs were found in coral larvae microbiomes, indicating that positive correlations with settlement were not a result of increased abundance of larvae-associated microbes (Tables S4-S7).

## Discussion

Coral larval settlement is an essential component of the coral recruitment process which contributes to sustaining healthy and resilient coral reefs [49]. While some settlement cues, or their sources, have been described [5], the majority remain uncharacterised, thereby limiting our understanding of settlement for a diverse range of coral species. Our study shows that marine biofilms developed in aquaculture can induce coral settlement, and that light exposure and development duration significantly affect biofilm composition and subsequently larval settlement. Further, we show that specific groups of taxa are consistently correlated with high or low coral settlement, suggesting these lineages have disproportionate influences on larval settlement through their associated inducing or inhibiting biochemistry. While these results have direct implications for improving coral settlement in aquaculture, importantly they also contribute to a better understanding of recruitment patterns among non-acroporid coral species, which is crucial for projecting future reef conditions under different climate scenarios [50].

### Environmental factors shape settlement inducing biofilms

The community composition of biofilms can impact larval settlement of marine invertebrates [50, 51], and here we show that light exposure and biofilm maturity are two key factors that influence the development of biofilms inductive of coral larval settlement. The 2-month (2 M) light treatment developed a markedly different biofilm community compared to the 2 M dark and 1-month (1 M) light conditioned biofilms and induced the highest levels of larval settlement for all coral species tested. This aligns with previous settlement results using artificial surfaces conditioned in the field, where 2-week-old biofilms induced less than 10% metamorphosis in *Acropora microphthalma,* while 8-week-old biofilms induced greater than 40% metamorphosis [12]. Additionally, higher coral settlement was observed on 8-week-old biofilms that formed at a depth of 4 m compared to 8 m, which may be related to light intensity. A similar study with *Acropora tenuis* larvae showed that 15-day-old biofilms induced higher coral settlement than 7-day-old biofilms, and that settlement increased on biofilms that were developed further away from mariculture sites with improved water quality [52]. Hence, along with biofilm maturity, environmental parameters such as light, depth and water quality can shift the community composition of biofilms thereby affecting recruitment on shallow-water coral reefs [53].

Our results demonstrated that even small changes in the biofilm community can impact larval settlement. For instance, the pre-existing microbial communities and biofilms in the experimental tanks systems influenced the composition of biofilms that formed on the settlement substrates, which in turn affected larval settlement. Therefore, despite controlling for factors such as light intensity and water temperature, each tank functioned as a separate system, introducing variability into the conditioning process. This suggests that minor shifts in biofilm development can have broader ecological implications for coral recruitment on reefs. Biofilms are likely to vary within and between habitats [54, 55], and this variability may influence coral recruitment patterns, ultimately shaping the community composition of reef corals. Further, since degraded reefs or those with poor water quality can have different biofilm communities compared to healthy reefs [53], this may promote a shift in coral community composition. In this study, *P. lobata* and *L. corymbosa* had less settlement on 2 M light treatment biofilms compared to other species, while *E. aspera* was the only species to show significant levels of settlement on dark treatment biofilms compared to controls. Hence, optimal biofilm conditions likely vary among coral taxa and this variation may correspond to the environmental conditions best suited to each species.

### A subset of biofilm taxa correlates with high or low settlement

Community composition differences between high and low settlement biofilms within treatment were less pronounced than community differences between treatments. Therefore, it is likely that changes in the abundance of a smaller group of microbes within these communities are driving coral settlement. ASVs classified as *Flavobacteriaceae* were positively correlated with high settlement in three of four coral species and were more abundant in the 2 M light biofilms compared to other treatments. The *Flavobacteriaceae* are key components of marine biofilms with genomes that encode a diverse range of secondary metabolite biosynthesis pathways [56, 57], and members of this family have previously been associated with settlement induction of marine invertebrate larvae. For example, biofilms that induce mussel settlement were treated with an antimicrobial agent, reducing the relative abundance of *Flavobacteriaceae* which correlated with a reduction in mussel settlement [58]. For coral, older biofilms that induced settlement of *A. microphthalma* larvae were associated with higher abundances of the *Cytophaga-Flavobacterium* group [12], while isolates of the *Cytophaga-Flavobacterium* group have shown high settlement induction of the polychaete *Hydroides elegens* [59]. Interestingly, high relative abundances of *Flavobacteriaceae* are associated with coral recruits of the species *Pocillopora acuta* in the first 1–2 weeks post-settlement [60]. Therefore, settlement on biofilms with specific taxa may be important for the uptake of early life stage symbionts.

Similarly, ASVs classified as *Pirellulaceae* and *Rhizobiaceae* were consistently correlated with high coral settlement across three different coral species, while some *Rhodobacteraceae* ASVs correlated with high settlement of *P. lobata* and *E. aspera*. The *Rhodobacteraceae* are abundant primary colonisers of marine biofilms and thought to be important for structuring communities into high-settlement biofilms [13, 61]. Further, an isolate of *Roseivivax* sp., within the *Rhodobacteraceae* family, was reported to induce larval settlement of the coral *Porites astreoides* [14], and this genus was found to correlate with high settlement for *Porites lobata* in this study. Hence, the *Rhodobacteraceae* family may be important for settlement of corals through both direct stimulation of settlement and biofilm community organisation*.* On the other hand, neither *Pirellulaceae* nor *Rhizobiaceae* have been implicated in the direct settlement of coral larvae and may represent new lineages for exploration. In particular, the phylum Planctomycetota (containing *Pirellulaceae*) has been observed to increase in abundance in coral reef biofilms as they develop over time [13, 55]. This suggests they are secondary colonisers and may be important members of mature biofilms that are more successful at inducing larval settlement. Although no difference was observed in *Rhizobiaceae* abundance between one- and two-month development times in this study, it has previously been associated with early biofilm colonisation [55].

Interestingly, many families that contained ASVs correlated with high settlement also contained other ASVs correlated with low settlement, and in some cases, this trend was observed at the genus level. For example, the genus *Winogradskyella* (*Flavobacteriaceae*), which was associated with low settlement for *P. sinensis* and high settlement for *P. lobata*, and the genus *Limibaculum* (*Rhodobacteraceae*), which was associated with both low and high settlement for *P. lobata*. Similar results have been observed with *Acropora tenuis* settlement, where some ASVs classified as the orders *Rhodobacterales* and *Flavobacteriales* had positive associations with coral settlement and others had negative associations [52]. This indicates that microbial inducers or inhibitors of coral larval settlement are species or even strain specific, and broad phylogenetic assignments are not predictive of inductive capacity. This has been observed in the genus *Pseudoalteromonas*, which can be a potent inducer of settlement or metamorphosis for a variety of marine invertebrate larvae including coral [20, 31, 62]. Yet closely related species of isolates that induce settlement have demonstrated vastly different effects on larval settlement, ranging from induction via multiple mechanisms, to no activity or even toxicity to larvae of some species [15, 17, 27]. Given the relatively high abundance and diversity of families such as *Rhodobacteraceae* and *Flavobacteriaceae* in marine biofilms, it is feasible they contain both inducing and inhibiting bacteria for coral settlement. Resolving strain level differences among potential inducers of larval settlement will be an important consideration when selecting prokaryotes for biochemical applications in restoration, and future studies would benefit from characterising settlement inducing biofilms beyond 16 s rRNA amplicons.

Although there was consistency at the family level for which taxa correlated with high settlement across different coral species, we did not find an ASV that was correlated with high settlement across all species tested. While a lack of significant correlation does not necessarily mean no inductive capacity, the results still suggest it is unlikely that there is a universal bacterium responsible for inducing settlement across a diverse range of corals. In such a scenario, certain bacterial taxa could be cultured to induce settlement of endangered or difficult to settle coral species that are targeted for restoration. Alternatively, different bacterial lineages may be capable of similar functions, such as the production of certain metabolites, and hence it may be the metabolic capability that is important for inducing settlement. Moreover, optimal larval settlement may be reliant on a community of organisms responsible for producing a range of biochemical cues that induce settlement for a diversity of reef corals [17]. Hence, future research would benefit from investigating the genomic capability and metabolite production of settlement inducing bacteria to better understand the mechanism of bacteria induced settlement.

### Chemical extracts of biofilms can induce coral larval settlement

Chemical extracts from 2 M light biofilms induced settlement for one of two species tested, revealing that coral larvae can respond to chemical cues within the biofilm in the absence of surface topography. Here, dichloromethane (DCM) extracts were more successful as a settlement inducer for *Porites lobata* compared to ethanol (EtOH) extracts, indicating hydrophobic/non-polar compounds may be more effective as settlement inducers for *P. lobata* than polar compounds*.* This result contrasts with most CCA-associated chemical inducers for the settlement of acroporid and agariciid larvae which are primarily soluble in EtOH or hot water [18, 24, 36]. Similarly, while both acroporid and non-acroporid corals were induced to settle by live CCA (*Porolithon onkodes*), only acroporid species settled in response to EtOH extracts [9]. This further indicates many non-acroporids may have a stronger settlement preference for hydrophobic compounds, with classes of chemical inducers differing between acroporid and non-acroporid clades. Interestingly, some bacteria may provide both soluble and hydrophobic compounds capable of settlement induction. For example, *Pseudoalteromonas* spp. are associated with both EtOH soluble compounds such as tetrabromopyrrole (TBP) [20] and hydrophobic compounds such as cycloprodigiosin [22]. Although the relative abundance of *Pseudoalteromonas* spp. was negligible within the biofilms of this study, it is possible that cycloprodigiosin or similar classes of hydrophobic inducers may be produced by other prokaryotes in the inductive biofilms.

The chemical extracts of biofilms did not induce settlement for *L. corymbosa*, and this coral also showed the weakest response to live biofilms. *L. corymbosa* has previously shown a different settlement response to certain species of CCA compared to the other corals tested here [8]. For example, the CCA *Porolithon* sp. induced > 50% settlement for all species tested here, except *L. corymbosa*, which had a mean of 28% settlement. On the other hand, *Sporolithon* sp. induced a mean of 87% settlement in *L. corymbosa* and 86% settlement in the closely related *E. aspera*, however only induced 45–58% settlement in *P. sinensis*, *D. favus* and *P. lobata* [8]. These different settlement cues might arise from different ecological preferences and life-history characteristics in the diverse species tested. For example, while corals from the genus *Lobophyllia* and *Echinophyllia* (Lobophyllidae) are known to be aggressive competitors for space [63], they occupy different niches on the reef, with *Echinophyllia* spp. more commonly found in shaded environments compared to *Lobophyllia* spp. [64]. Species-specific larval responses to different settlement cues are likely to be linked to the recognition of microbial communities or CCA associated with preferred habitats, which in turn influences the spatial distribution of corals on the reef [65].

### Future directions

Exploring the association of biofilms with coral larval settlement offers promising opportunities for the discovery of natural biochemical inducers; however, research is still needed to understand how complex biofilms interact with coral larvae (Randall et al., 2020). Developing biofilms under dark conditions is likely to have reduced the contribution to inductive biochemistry by eukaryotes such as CCA spores or microalgae, and moderate settlement induction of *E. aspera* larvae was observed on these biofilms. This supports the potential role of non-phototrophic prokaryotes in the settlement of non-acroporid larvae; however, the greatest settlement was observed in response to 2 M light biofilms. Hence, it is possible that phototrophic eukaryotes may have contributed to inductive biochemistry in this treatment. Fully disentangling the role(s) of prokaryotes and eukaryotes in triggering coral larval settlement requires assessing the inductive capacity of isolates from biofilm taxa [16, 17, 20], and future research may benefit from targeting some of the taxa identified here. Genome sequencing paired with comparative analyses of inductive and non-inductive isolates could help reveal the molecular machinery underpinning settlement induction. However, challenges remain as most prokaryotes are not readily cultured and their biochemistry in isolation is likely to differ from that in situ [66]. Nonetheless, controlling the settlement of non-acroporids in aquaculture using biochemical inducers from cultured prokaryotes may be more practical compared to induction by CCA as it eliminates the need to continually harvest specific algal species from the reef and has potential for large-scale standardised production.

## Conclusions

Despite the importance of coral recruitment and decades of research, we still lack fundamental knowledge on the identification of taxa and mechanisms that underpin microbially induced coral larval settlement. Our research shows that biofilm development is integral to the success of larval settlement and, therefore, plays an important role in the recovery of coral reefs. Additionally, we show that certain lineages of bacteria are consistently correlated with coral settlement, such as those classified as *Flavobacteriaceae* and *Rhodobacteraceae*. Although no single universal inducer was identified, similar taxa may share functional traits leading to the production of similar biochemical cues. This knowledge offers a platform to target, isolate and begin experimentally testing these lineages for their ability to induce settlement, as well as obtaining genomic resources to address the mechanisms behind settlement induction. Resulting biochemical applications to increase coral settlement can be implemented in restoration programs worldwide.

## Supplementary Information

Additional file 1.

Additional file 2.

Additional file 3.

Additional file 4.

Additional file 5.

Additional file 6.

Additional file 7.

## Data Availability

All sequence data generated and analysed in this study are available at the NCBI Sequence Read Archive (SRA) (https://www.ncbi.nlm.nih.gov/sra) under the BioProject ID PRJNA1107340. All code and associated data files used in the analyses are available at https://github.com/paobrien/Microbial-inducers-of-coral-settlement. Reviewer link for sequence data: https://dataview.ncbi.nlm.nih.gov/object/PRJNA1107340?reviewer=cesobm45ms50huaqv0fcfa953n
