# Supplementary material for: Light and dark biofilm adaptation impacts larval settlement in diverse coral species
Source: Environ Microbiome. 2025 Jan 25;20:11. doi: 10.1186/s40793-025-00670-0 (PMC11762876; doi:10.1186/s40793-025-00670-0)
Supplement: Supplementary file 1 — Additional file 1. [file 40793_2025_670_MOESM1_ESM.docx]

**Light and dark biofilm adaptation impacts larval settlement in diverse coral species**

Paul A. O’Brien, Sara C. Bell, Laura Rix, Abigail C. Turnlund, Shannon R. Kjeldsen, Nicole S. Webster, Andrew P. Negri, Muhammad Abdul Wahab, Inka Vanwonterghem

**Supplementary Figures and Tables**

**Figure S1.** Biofilm conditioning and experimental setup of light and dark treatments. Top panel shows the dark and light conditioning tanks inside the larger fibreglass tank. Concrete settlement tabs can be seen conditioning on the right-side tank with two time points, 1 month (front) and 2 months (back) before spawning. Bottom panel shows a closeup of concrete settlement tabs with biofilm development.

**Figure S2.** Settled coral larvae undergoing metamorphosis, characterised by flattening of the larvae post attachment and the development of an oral disc and septae. A) *P. sinensis*, B) *Dipsastrea favus,* C) *E. aspera,* D) *P. lobata* and E) *L. corymbosa*. Panel F) shows two planula larvae for *P. sinensis*, one elongated and one rounded. All settlement photos are in response to 2M light conditioned biofilm, except *P. lobata*, which is in response to EtOH chemical extract. Scale bar for settlement images = 1mm, scale bar for planula larvae = 500µm. Images were taken using a stereo microscope fluorescence adaptor (https:// night sea. com/; SFA RB – excitation 440–460 nm, emission filter 500 nm longpass) that excites the larval green fluorescent proteins. Images of coral larvae taken under white light.

**Figure S3. November assays.** Proportion of coral larvae settled in response to DCM and EtOH chemical extracts of volumes 5, 10, 25, 50, 100 µL. GLM results showed that *P. lobata* settlement was significantly lower in response to the EtOH extract compared to DCM (*coef* = -0.86, *z* = -2.21, *p* = 0.025).

**
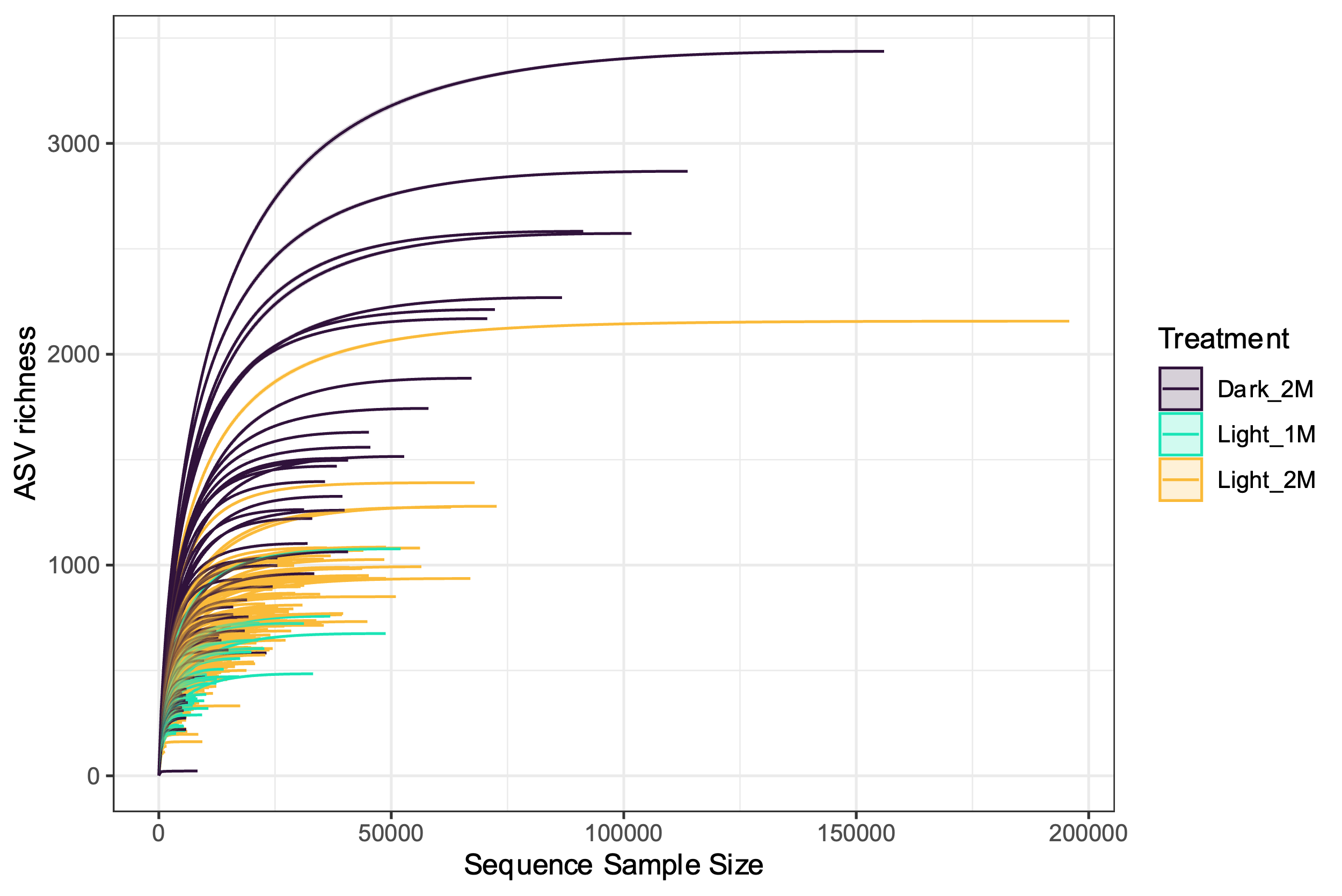
**

**Figure S4.** Rarefaction plot illustrating the number of ASVs against sequencing depth for each sample coloured by treatment. Samples from all treatment groups reached asymptote, indicating the full diversity of ASVs has been captured, except two samples from the 2-month light treatment that contained less than 4000 reads.

A)


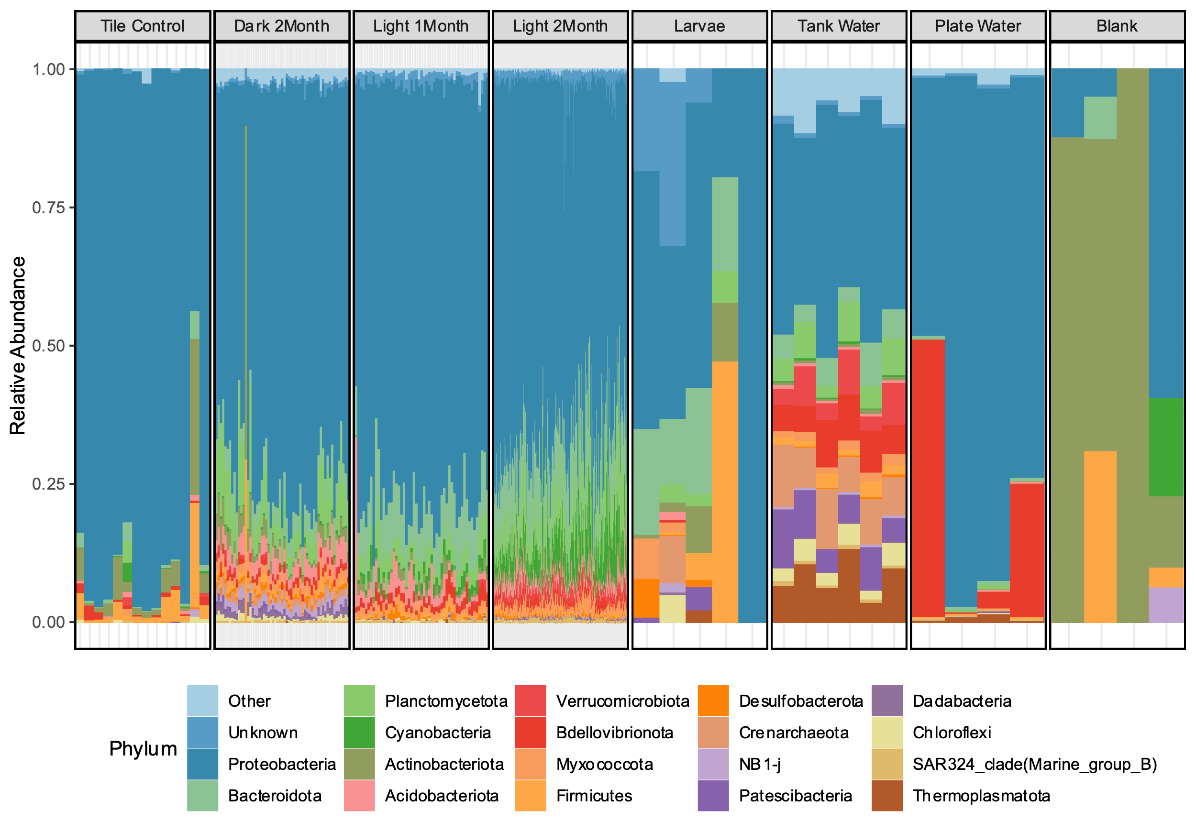


**
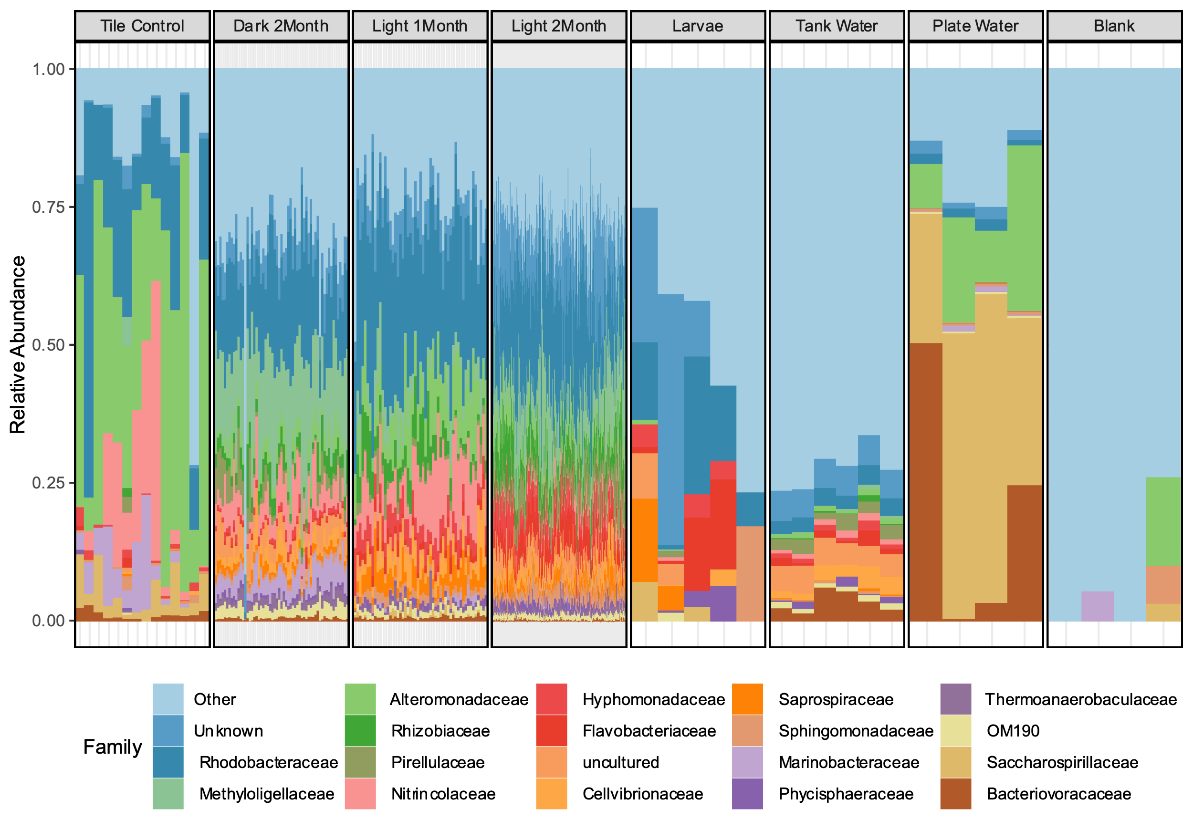
**B)

**Figure S5.** Relative abundance of the top 20 most abundant prokaryote A) phyla and B) families across all biofilm samples and controls including unconditioned settlement tabs (control), larvae samples, conditioning tank water, assay plate water and extraction blanks. Profiles of control samples differ substantially from those of biofilms.

A)


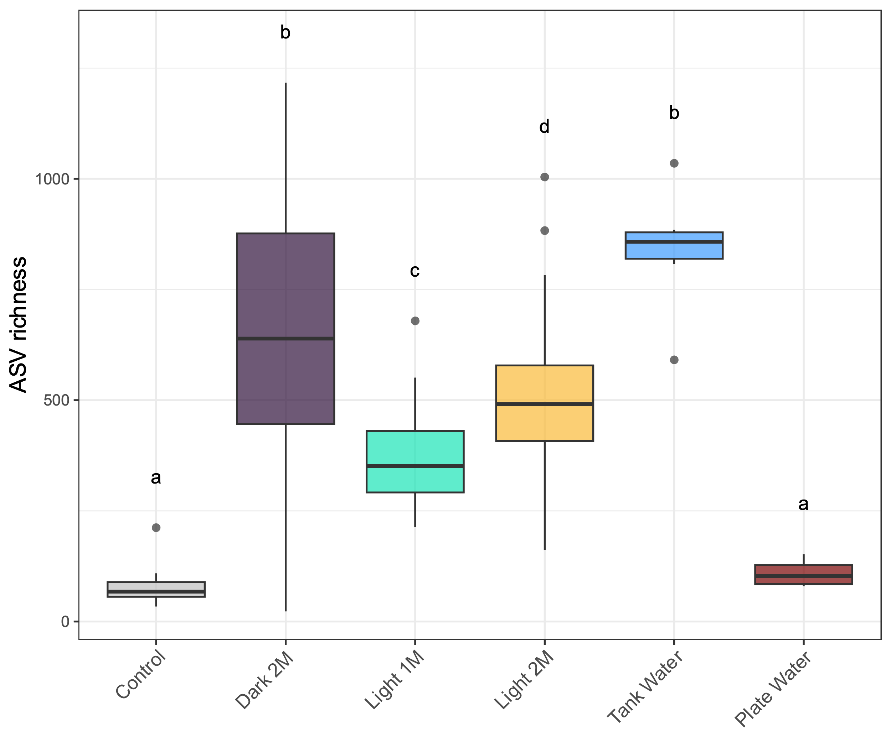


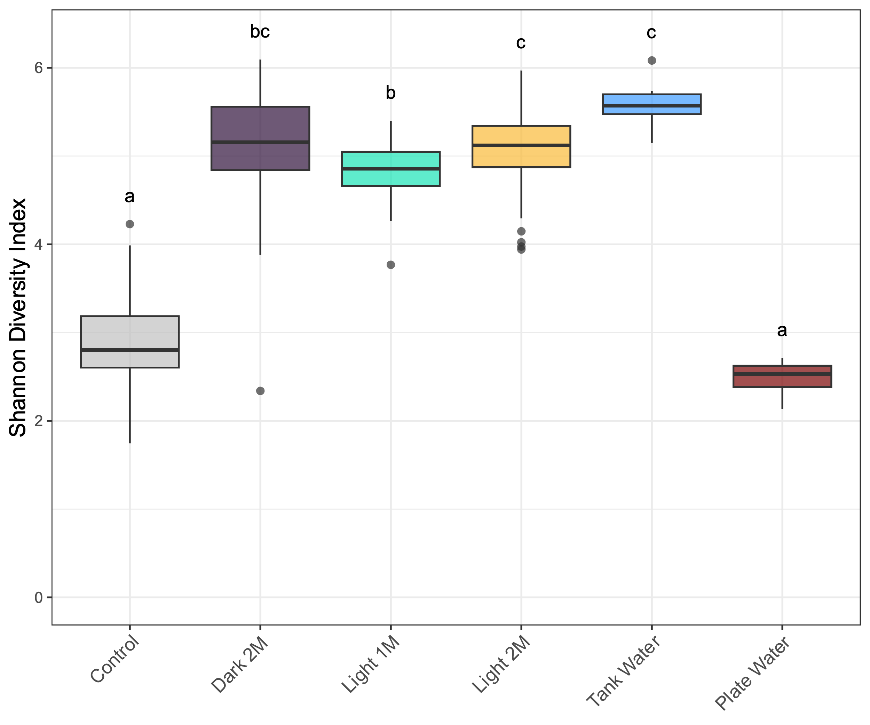
B)

**Figure S6.** ASV richness (A) and Shannon Diversity Index (B) for biofilms in each conditioning treatment and controls including unconditioned settlement tabs (Control), conditioning tank water and assay plate water. Larvae and blank controls were removed during rarefaction due to the low number of sequences. Letters denote which treatments were significantly different from each other (*p* < 0.05).

**
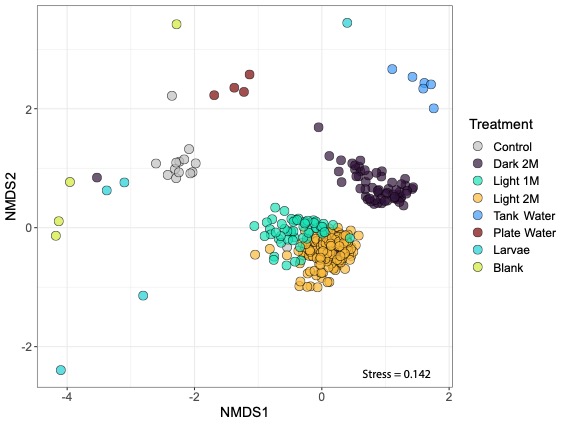
**A)

**
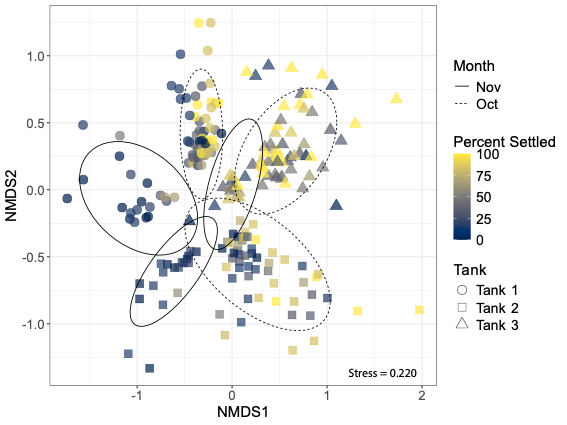
**B)

**Figure S7.** Bray-Curtis dissimilarity visualised using NMDS for A) Biofilms across all treatments and control samples including unconditioned settlement tabs (control), conditioning tank water, assay plate water, larvae and extraction blanks. Biofilm samples are coloured by treatment, and B) Biofilms from the 2M light conditioned treatment with ellipses indicting the conditioning month separated by conditioning tank (shape). Biofilm samples are coloured by the percent of larvae settled.

**Supplementary Tables**

**Table S1.** Summary statistics and GLMM fixed effects table. Summary statistics are based on percentage of corals settled in each treatment. GLMM fixed effects table is based on the model fit by maximum likelihood (Laplace Approximation) with a binomial distribution. All values represent treatments compared to the intercept (control samples). Model formula: cbind(Total_settled, Total_not_settled) ~ Treatment + (1 | Tank/Sample_ID)

**Table S2**. PERMANOVA results when analysing all conditioning treatments. Factors include conditioning treatment, settlement (high, med, low categories), conditioning tank and coral species.

**Table S3.** PERMANOVA results when analysing only 2-month light conditioning treatment. Factors including conditioning tank, settlement (high, med, low categories) and coral species

**Tables S4-S7.** ASVs identified as potential inducers or inhibitors of coral settlement for *P. sinensis* (S4), *D. favus* (S5), *E. aspera* (S6), and *P. lobata* (S7). Columns A, B and Group refer to A/B statistics from the indicator species (IS) analysis and whether they were associated with high or low settlement. LM Coef refers to the linear model (LM) coefficient where a positive value is a positive correlation with settlement. RF importance refers to the relative importance for predicting settlement in the random forests (RF) analysis. Only the top 20 ASVs were included, and only those additionally found in either the IS or LM analyses, since the RF value does not indicate high or low settlement. Finally, present in controls depicts if the ASV was also present in control samples for larvae (LV), extraction blanks (EB), negative substrate control (Neg), tank water (TW) and assay plate water (PW).

*Table legend only - tables uploaded separately*

**Table S8**. Mean relative abundance and standard deviation for the total sum of putative inducing and inhibiting ASVs for coral larval settlement in each treatment. Light 2M and control biofilms represent biofilms used to settle the listed species. Light 1M and Dark 2M biofilms were used for the settlement of *E. aspera* larvae
