# Supplementary material for: Light and dark biofilm adaptation impacts larval settlement in diverse coral species
Source: Environ Microbiome. 2025 Jan 25;20:11. doi: 10.1186/s40793-025-00670-0 (PMC11762876; doi:10.1186/s40793-025-00670-0)
Supplement: Supplementary file 2 — Additional file 2. [file 40793_2025_670_MOESM2_ESM.docx]

**Light and dark biofilm adaptation impacts larval settlement in diverse coral species**

Paul A. O’Brien, Sara C. Bell, Laura Rix, Abigail C. Turnlund, Shannon R. Kjeldsen, Nicole S. Webster, Andrew P. Negri, Muhammad Abdul Wahab, Inka Vanwonterghem

**Supplementary file 1 - Methods**

*Chemical extraction of biofilms*

To understand if chemical compounds from biofilms on the conditioned sheets would induce settlement, we performed chemical extractions on a subset of our 2M light treatment biofilms prior to spawning using two solvents: 1) ethanol (EtOH) for polar compounds and 2) dichloromethane (DCM) for hydrophobic compounds. For both the EtOH and DCM extractions, 15 conditioned tabs (14×14 mm) were collected from each replicate experimental tank and pooled, resulting in 45 tabs total. Settlement tabs were first rinsed in reverse osmosis (RO) purified water to remove salts and those required for the DCM extraction were freeze-dried. Tabs were placed in a 250 mL Schott bottle pre-cleaned with acetone and 100 mL of either EtOH or DCM was added for extraction. Schott bottles were sonicated using a sonicator bath for 80 min and the solvent decanted into a new pre-cleaned 250 mL Schott and stored at -20°C. The extraction process was repeated by adding another 100 mL of either EtOH or DCM to the Schott bottles containing settlement tabs and sonicated for a further 80 min. Supernatants of the same solvent were combined and then filtered through a filter paper (Whatman; Grade 2, 8 µm) into a 500 mL round bottom flask, followed by rotatory evaporation and freeze-drying. Extracts were dissolved using EtOH followed by DCM (for both extraction solvents) and transferred to a pre-weighed scintillation vial. The volume of each vial was reduced using a stream of N_2_, freeze-dried and then weighed to calculate the mass of residue. Each extract was resuspended in 4.5 mL of EtOH (proportional to 0.1 mL per settlement tile) and stored at -20°C. To create a negative control treatment, unconditioned settlement tabs (negative controls as described above) were extracted in parallel using the same method. The final residue mass of each extraction was 0.222 g, 0.1618 g, 0.1549 g and 0.1293 g for DCM 2M-light, DCM unconditioned, EtOH 2M-light and EtOH unconditioned respectively.

*DNA extraction and sequencing*

Biofilm and larval samples were thawed on ice and DNA was extracted using the DNeasy UltraClean Microbial Kit (Qiagen) following the manufacturer’s protocol. Blank extraction controls were extracted in parallel following the same protocol in the absence of a sample. Sterivex filters containing water samples were thawed and DNA extracted using a Phenol:Chlorofom:Isoamyl Alcohol protocol detailed in Botté et al., 2019, with the addition of 18µl of 100mg/ml lysozyme to the lysis buffer and initial incubation at 37°C for one hour. DNA was quantified using a Qubit fluorometer, quality checked using a NanoDrop spectrophotometer (ThermoFisher) and checked for PCR amplification prior to sequencing.

16S rRNA amplicon sequencing (2 x 300bp) was conducted at the Australian Centre for Ecogenomics (ACE) on the Illumina MiSeq (v3) platform using the modified V4 region primer set, 515F (GTGYCAGCMGCCGCGGTAA) (Parada et al., 2016) and 806R (GGACTACNVGGGTWTCTAAT) (Apprill et al., 2015). Preparation of the 16S library was performed following the Illumina # 15044223 Rev. B protocol with an alteration in polymerase used to substitute NEBNext® Ultra™ II Q5® Mastermix (New England Biolabs #M0544) in standard PCR conditions.

**Buffers and reagents use to process biofilms from settlement tabs**

**Cell separation buffer**

350mg PVP

133.9mg Sodium pyrophosphate

To 100ml with CMFSW

Dissolve by mixing and warming and split into 2 x 100mL schott bottles to autoclave

Add 500ul Tween 20 after autoclaving – mix gently

**Lysis buffer**

5 mL 1 M Tris-HCl (pH 8.0)

8 ml 0.5 M EDTA (pH 8.0)

25.6 g Sucrose

Make up to 100 mL with MQ Water

Filter Sterilize (0.22 µm) into 2 x 50 mL falcon tubes

**Lysozyme**

Add 0.1 g (100 mg) lysozyme powder to 1 mL MQ Water (100 mg/mL)

**References**

Apprill, A., Mcnally, S., Parsons, R., & Weber, L. (2015). Minor revision to V4 region SSU rRNA 806R gene primer greatly increases detection of SAR11 bacterioplankton. *Aquatic Microbial Ecology*, *75*(2), 129–137. https://doi.org/10.3354/ame01753

Botté, E. S., Nielsen, S., Abdul Wahab, M. A., Webster, J., Robbins, S., Thomas, T., & Webster, N. S. (2019). Changes in the metabolic potential of the sponge microbiome under ocean acidification. *Nature Communications*, *10*(1), 1–10. https://doi.org/10.1038/s41467-019-12156-y

Parada, A. E., Needham, D. M., & Fuhrman, J. A. (2016). Every base matters: Assessing small subunit rRNA primers for marine microbiomes with mock communities, time series and global field samples. *Environmental Microbiology*, *18*(5), 1403–1414. https://doi.org/10.1111/1462-2920.13023
