# Supplementary material for: Light and dark biofilm adaptation impacts larval settlement in diverse coral species
Source: Environ Microbiome. 2025 Jan 25;20:11. doi: 10.1186/s40793-025-00670-0 (PMC11762876; doi:10.1186/s40793-025-00670-0)
Supplement: Supplementary file 3 — Additional file 3. [file 40793_2025_670_MOESM3_ESM.docx]

**Light and dark biofilm adaptation impacts larval settlement in diverse coral species**

Paul A. O’Brien, Sara C. Bell, Laura Rix, Abigail C. Turnlund, Shannon R. Kjeldsen, Nicole S. Webster, Andrew P. Negri, Muhammad Abdul Wahab, Inka Vanwonterghem

**Supplementary file 2 – Results**

*Taxonomic profile of biofilms developed under different conditioning treatments*

Of 69 prokaryotic phyla found, the Proteobacteria had the highest relative abundance with a mean of 68.5 ± 9.3%, followed by Bacteroidota and Planctomycetota with a mean of 7.8 ± 3.4% and 7.3 ± 3.8% respectively (Figure 2a; Figure S4a). Cyanobacteria were most abundant in the 2M light treatment (5.3 ± 3.8%) compared to the 1M light (1.5 ± 1%) and 2M dark (0.4 ± 0.6%) treatments. Similarly, the Bacteroidota were more abundant in the 1M light (8.1 ± 3.0%) and 2M light (9.0 ± 2.7%) treatments compared to the 2M dark (3.0 ± 0.9%). On the other hand, Crenarchaeota had a relative abundance of 1.3 ± 0.8% in the 2M dark conditioned biofilms but were nearly absent from the 1M light (0.01 ± 0.05%) and 2M light (0.01 ± 0.04%) conditioned biofilms. Finally, the Dadabacteria were more abundant in the 2M dark treatment (1.1 ± 0.7%) compared to the 1M light (0.03 ± 0.05%) and 2M light (0.2 ± 0.2%) treatments.

At the family level, *Rhodobacteraceae* was the most abundant across all treatments (19.6 ± 7.5%), followed by *Methyloligellaceae* (8.7 ± 6.9%) and *Rhizobiaceae* (4.8 ± 2.9%) (Figure S4b). The *Flavobacteraceae* were more abundant in biofilms from the 2M light treatment (4.1 ± 2.0%) compared to the 1M light (1.5 ± 0.9%) and 2M dark (0.4 ± 0.4%) treatments, while the *Hyphomonadaceae* were more abundant in both 1M light (3.5 ± 1.3%) and 2M light (3.6 ± 2.1%) compared to the 2M dark (0.8 ± 0.5%) biofilms. Conversely, *Methyloligellaceae* were more abundant in the 2M dark treatment (15.7 ± 5.7%) compared to the 1M light (3.3 ± 2.8%) and 2M light (8.1 ± 6.3%) biofilms. Finally, *Marinobacteraceae* had a relative abundance of 4.2 ± 1.8% in the 2M dark treatment, yet was mostly absent from the 1M light (0.5 ± 0.7%) and 2M light (0.5 ± 0.4%) biofilms.

*Alpha and beta diversity of biofilm communities across conditioning treatments*

Estimates of ASV richness were significantly different among conditioning treatments (ANOVA; *F* = 28.4_(2,315)_, *p* < 0.001), where 2M dark biofilms encompassed the largest mean ASV richness (659 ± 276 SD), followed by 2M light (493 ± 138 SD) and 1M light (367 ± 104 SD) biofilms at a sampling depth of 4000 reads (Figure 2b; Figure S5a). Shannon Diversity Index (*H*), which considers the number of ASVs as well as their abundances, similarly found a significant difference in diversity among conditioning treatments (ANOVA; *F* = 6.6_(2,315)_, *p* = 0.002). However, post hoc analyses revealed no difference in diversity between the 2M light (mean *H =* 5.09 ± 0.38 SD) and 2M dark (mean *H =* 5.13 ± 0.61 SD) biofilms, which both had a higher diversity than the 1M light biofilms (mean *H =* 4.83 ± 0.32 SD; Figure 2c; Figure S5b). Hence, although dark treatment biofilms had the highest ASV richness, these biofilms likely contain a larger number of rare ASVs with low abundances compared to light treatment biofilms.
